# Tissue-resident memory T cells in the kidney

**DOI:** 10.1007/s00281-022-00927-7

**Published:** 2022-04-11

**Authors:** Nariaki Asada, Pauline Ginsberg, Nicola Gagliani, Hans-Willi Mittrücker, Ulf Panzer

**Affiliations:** 1 III. Department of Medicine, Division of Translational Immunology, University Medical Center Hamburg-Eppendorf, Hamburg, Germany; 2 Hamburg Center for Translational Immunology (HCTI), University Medical Center Hamburg-Eppendorf, Hamburg, Germany; 3 Department of General, Visceral and Thoracic Surgery, University Medical Center Hamburg-Eppendorf, Hamburg, Germany; 4 I. Department of Medicine, University Medical Center Hamburg-Eppendorf, Hamburg, Germany; 5 Immunology and Allergy Unit, Department of Medicine, SolnaKarolinska Institute and University Hospital, Stockholm, Sweden; 6 Institute for Immunology, University Medical Center Hamburg-Eppendorf, Hamburg, Germany

**Keywords:** T cell, Tissue-resident memory T cell, Infection, Autoimmune kidney disease

## Abstract

The identification of tissue-resident memory T cells (T_RM_ cells) has significantly improved our understanding of immunity. In the last decade, studies have demonstrated that T_RM_ cells are induced after an acute T-cell response, remain in peripheral organs for several years, and contribute to both an efficient host defense and autoimmune disease. T_RM_ cells are found in the kidneys of healthy individuals and patients with various kidney diseases. A better understanding of these cells and their therapeutic targeting might provide new treatment options for infections, autoimmune diseases, graft rejection, and cancer. In this review, we address the definition, phenotype, and developmental mechanisms of T_RM_ cells. Then, we further discuss the current understanding of T_RM_ cells in kidney diseases, such as infection, autoimmune disease, cancer, and graft rejection after transplantation.

## Introduction

The kidney is a unique non-barrier organ that can be challenged by different types of immune-associated pathologies such as infections, autoimmune diseases, cancer, and graft rejection after kidney transplantation. In these conditions, different types of immune responses are involved that lead to either resolution or progression of the disease. Tissue-resident immunity can be immediately activated on the inflammation site and has important roles in the early phase of immune reactions (1). Adaptive immunity by T cells and B cells contributes to an antigen-specific and efficient immune reaction (2). However, upon the first antigen challenge, adaptive immunity takes longer to be activated as compared with the innate immune system [1, 2]. One major advantage of adaptive immunity is immune memory formation, which enables a swift response upon repeated antigen challenge [1–3]. In the last decade, tissue-resident memory T cells (T_RM_ cells), a new immune cell population, have been characterized as an *antigen-specific frontline defense* in peripheral organs [4]. T_RM_ cells can, immediately and efficiently, fight specific antigens. Most important, recent findings suggest that T_RM_ cells not only have roles in the host defense but also in unwanted inflammation such as autoimmune disease [4].

## General background of TRM cells

The protection of peripheral tissues from invading pathogens and microbes is a central function of the adaptive immune system. Upon challenge, antigens of the pathogens are taken up by dendritic cells (DC) [1]. In response to inflammatory signals, dendritic cells (DC) migrate to secondary lymphoid tissue and present processed antigens via MHC class I or II to CD8^+^ or CD4^+^ T cells, respectively. After antigen recognition, T cells become activated, start to proliferate, and differentiate into effector T cells, which migrate to the site of inflammation [1]. Ideally, this response results in the elimination of the non-self-antigen and, ultimately, resolution of the local inflammation. As a consequence, the antigen-specific T-cell population contracts. However, a subset remains as a memory T-cell population, which is responsible for an accelerated response when re-encountering the antigen.

Until recently, memory T cells were divided into two main groups, namely effector memory T cells (T_EM_ cells) and central memory T cells (T_CM_ cells) [3, 5]. T_EM_ cells can enter peripheral tissues from the blood vessels, migrate via lymphatic vessels to secondary lymphoid organs, and eventually re-enter the blood stream [6]. Upon recurring antigen challenge, activated T_EM_ cells are responsible for the accelerated T-cell response at peripheral sites. In contrast, T_CM_ cells are mainly found in the lymphoid tissue and blood. T_CM_ cells have a strong proliferative capacity and are thought to support and replenish T_EM_ cells [3]. However, the concept of memory T cells has dramatically changed by the identification of T_RM_ cells, a memory T-cell population that persists in tissue after resolution of the acute T-cell response.

The identification of T_RM_ cells as a novel memory T-cell population was mainly achieved by innovative technical approaches, namely parabiosis and intravenous labeling. Parabiosis, the surgical connection of the blood circulation of two mice, revealed the exclusion of a population of memory T cells from the circulation and their residency in peripheral tissues for extended time periods [7, 8]. After several weeks of parabiosis, circulating T-cell populations reach an equilibrium. In contrast, a substantial memory T-cell population in peripheral tissues fails to reach this equilibrium and remains in the original tissue [7, 8]. Tissue residency of T_RM_ cells is also observed in tissue transplantation models in which a large population of donor T cells persists in the graft tissue for months [8]. A further novel approach was the development of in vivo labeling techniques for intravascular T cells. After intravenous injection, fluorochrome-conjugated antibodies require several minutes to cross blood vessel endothelia. If antibodies are injected shortly before cell isolation, intravascular cells can be identified by the presence of antibody staining, leaving T_RM_ cells and other extravascular T cells unstained [9, 10]. Together, these approaches allowed the isolation and in-depth characterization of T_RM_ cells in mouse models to be performed and revealed that T_RM_ cells significantly differ in phenotype, gene expression profile, and function from circulating memory T-cell subsets. Based on their characteristic phenotype and mRNA expression profile, T_RM_ cells were subsequently identified in human tissues, including barrier sites and internal organs including the kidney [4, 11, 12]. The identification of donor-derived T_RM_ cells in transplanted human tissues confirmed the long-term tissue residency of these cells and their exclusion from the recipients’ blood circulation [13, 14].

## Analysis of TRM cells in the kidney

Analysis of T_RM_ cells is usually based on flow cytometry, which requires harsh isolation procedures of cells from peripheral tissues and often results in the underestimation of T_RM_ cell numbers. In particular, renal T_RM_ cells are more difficult to isolate compared with intravascular T cells. The quantification of renal T_RM_ cells using immunofluorescence microscopy revealed that the numbers were 20 times higher as compared to those determined by the use of flow cytometry-based methods [10].

Under homeostatic conditions, only a small number of memory T cells can be isolated from non-lymphoid tissues of mice. This is mainly because laboratory mice are usually kept under specific pathogen- free conditions, with very limited challenge to their immune system. When freely living feral mice or pet store mice are analyzed, significantly increased numbers of T cells can be isolated from peripheral tissues, including the kidney [15]. The number of T cells in non-lymphoid tissue increases when laboratory mice are co-housed with pet store mice, indicating an important role for the environment. Under homeostatic conditions, a small number of T_RM_ cells can be isolated from the kidney, and a large fraction of these cells are CD8^+^ T_RM_ cells [9, 16]. The number of T_RM_ cells in mice kidney can be significantly increased using bacterial or viral infection models.

In the human healthy kidney, CD4^+^ T_RM_ cells are more abundant than CD8^+^ T_RM_ cells [17, 18]. Compared to barrier organs such as the intestine, the number of T_RM_ cells in healthy kidney is low. Nevertheless, in our experience, more than 10,000 CD4^+^ T_RM_ cells can be isolated from one gram of healthy human kidney tissue using fluorescence-activated cell sorting (FACS) (unpublished data).

## Surface markers of TRM cells in the kidney

There are currently only a few published studies addressing the phenotype of renal T_RM_ cells in humans. This is mainly because of limited access to healthy human tissue. In recent studies, healthy tissue from surgically removed kidneys due to tumor, or tissue from explanted renal allografts after transplant failure, was analyzed [17, 18]. Comparable to T_RM_ cells in other human organs, or in mouse kidneys, healthy human kidneys harbor T_RM_ cells with a similar surface phenotype.

T_RM_ cells are generally identified by the expression of CD69, a membrane-bound, type II C-lectin receptor [9]. CD69 inhibits expression of the sphingosine-1-phosphate receptor 1 (S1PR1), which is required for the egress of T cells from peripheral tissues [19, 20]. Although CD69 was originally described as an early activation marker for T cells, resting T_RM_ cells do not show an activated phenotype [21]. Other common markers for T_RM_ cells are CD103 and CD49a, which are subunits of the integrins αEβ7 and α1β1, respectively. CD103 and CD49a bind to adhesion molecules commonly expressed on epithelial cells, leading to tissue adherence of T cells [22, 23]. CD103 is a marker for CD8^+^ T_RM_ cells in mucosal tissues and skin [24, 25], but its expression is relatively rare in renal T_RM_ cells [23]. In the kidney, CD49a is a more reliable T_RM_ cell marker, especially for CD8^+^ T cells [22]. The chemokine receptors CXCR3 and CXCR6 are also frequently expressed on renal T_RM_ cells, which is comparable to findings in other non-lymphoid organs [17, 18, 26, 27]. Moreover, a recent study showed that CD8^+^ T_RM_ cells downregulate interleukin-18 receptor (IL-18R) during their development in the kidney [28]. Other general phenotypes of T_RM_ cells are S1PR1^low^, CD44^+^ (memory T-cell marker), CD62L^−^ (L-selectin, a cell adhesion molecule for homing to lymph tissue), and CCR7^−^ (chemokine receptor for lymphoid tissue recruitment) [29].

## Development of TRM cells in the kidney

An accumulation of pathogen-specific renal CD8^+^ T_RM_ cells was demonstrated in several mouse infection models [10, 30–32]. Most of the used pathogens, e.g., *Listeria monocytogenes* or lymphocytic choriomeningitis virus (LCMV), infect the kidney, at least transiently. After clearance of the infection, pathogen-specific T cells, together with the phenotype of T_RM_ cells, can be identified in the kidney and persist in the tissue for months. The evidence of tissue residency of these cells was also provided using parabiosis [18, 33].

CD69 expression is crucial to T_RM_-cell development in the kidney of mice. In CD69-deficient animals, the number of renal T_RM_ cells is significantly decreased [34], and expression of mutant CD69 which cannot interact with S1P1 fails to rescue the poor accumulation of T_RM_ cells in the kidney, suggesting that the interaction of CD69 with S1P1 is required for T_RM_-cell induction.

TGF-β induces the expression of CD103 during T_RM_-cell development [30, 35]. Although only a minor subset of renal CD8^+^ T_RM_ cells is CD103^+^ [36, 37], the accumulation of renal T_RM_ cells depends on TGF-β. In the absence of TGF-β signaling, T cells show an impaired upregulation of the P- and E-selectin ligands and reduced CXCR3 expression, which results in insufficient recruitment and transendothelial migration of T cells into the kidney [30].

IL-15 was demonstrated to be required for T_RM_-cell survival in tissues such as skin, lung, liver, salivary gland, and kidney, but it is dispensable for cells in the pancreas, female reproductive tract, and small intestine [38–40].

## Transcription profile of TRM cells

In addition to the analyses of surface markers, T_RM_ cells have been extensively characterized for their mRNA expression profile, and core expression signatures have been defined for both mouse and human T_RM_ cells. The signatures define T_RM_ cells as a unique cell population distinct from other T-cell subsets [24, 27, 41]. Furthermore, transcriptional heterogeneity exists within the T_RM_-cell population, which probably reflects different adaptations to the local environment, or consequences of the type of infection responsible for the formation of these cells [42].

Transcription factors regulating T_RM_-cell development include Hobit (encoded by the gene *Znf683*), Blimp-1 (encoded by *Prdm1*), and Runx3 [36, 43, 44]. The related transcription factors Hobit and Blimp-1 contribute to T_RM_-cell development in both CD4^+^ and CD8^+^ T cells. Hobit represses the expression of *Klf2*, which is required for tissue egress receptor *S1pr1* expression. Both Hobit and Blimp-1 bind to target sequences and suppress the expression of S1PR1, CCR7, and TCF-1, which are important for tissue egress [36]. In response to the interaction with ligands on cells from the environment, the transcription factor NOTCH controls the maintenance of CD8^+^ T_RM_ cells in peripheral tissue [41]. Furthermore, the transcription factor Bhlhe40 is important to facilitate the metabolic adaptation of T_RM_ cells for the maintenance of mitochondrial fitness [45].

Tissue residency also requires metabolic adaptation to nutrients and oxygen availability in the environment. Although it is unclear how renal T_RM_ cells adapt to the environment, previous studies showed upregulation of the hypoxia-inducible factor pathway in hepatic T_RM_ cells [46]. Given the hypoxic environment in the kidney [47, 48], it is likely that renal T_RM_ cells also upregulate the expression of hypoxia-responsive genes. In addition, an adaptation of the nutrient metabolism is crucial to the maintenance of T_RM_ cells. In CD8^+^ T_RM_ cells generated by viral infection in the skin, several molecules that mediate lipid uptake are highly expressed. In particular, the fatty acid binding proteins (FABP) 4 and 5 are essential to the exogenous uptake of free fatty acids (FFA), long-term survival, and even to T_RM_-cell functions [49].

## TRM cells in clinical settings

In this part, we will discuss T_RM_ cells in the human and mice kidney by focusing on their clinical relevance.

## TRM cells in kidney infection

Infection is the key trigger for T_RM_-cell induction in many different organs, including the kidney. Upon infection, T cells with cognate T-cell receptor (TCR) are activated and recruited to the infection sites. Some T cells remain in the organ as T_RM_ cells after resolution of the inflammation, working as sentinels that rapidly respond when re-encountering the pathogen. In murine model studies, the presence of T_RM_ cells significantly reduced the pathogen burden and protects mice from lethal challenge infections [50, 51].

**Fig. 1:**
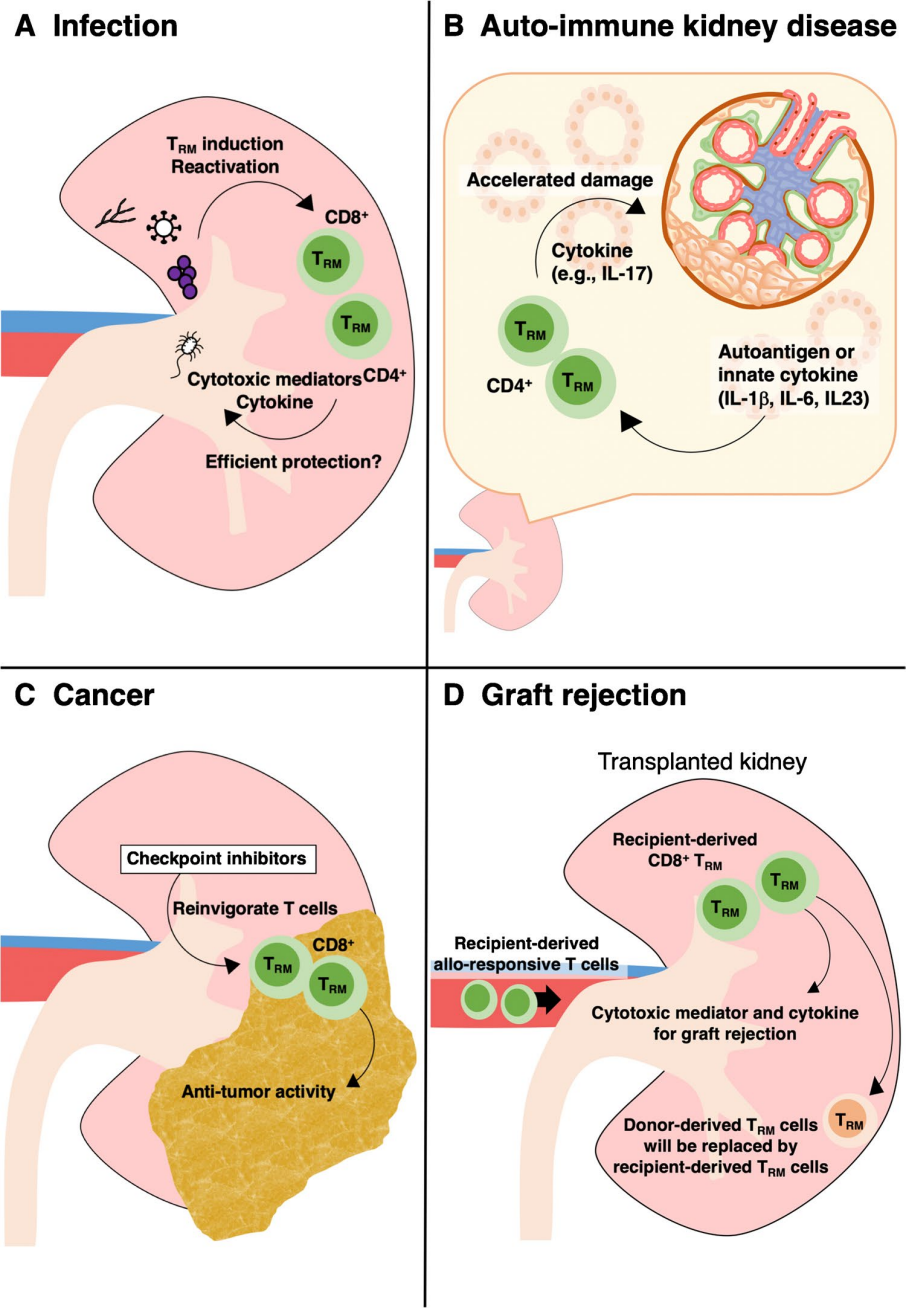
Role of T_RM_ cells in kidney diseases. **A**) Infection by pathogens such as bacteria, fungi, and virus, induces CD4^+^ and/or CD8^+^ T_RM_ cells in the kidney. They produce cytokines and cytotoxic mediators and are expected to contribute to efficient host defense. **B**) In autoimmune kidney disease, T_RM_ cells are activated by autoantigen and/or innate cytokines, and aggravate disease by producing inflammatory cytokines. **C**) In renal cancer such as renal cell carcinoma, CD8^+^ T cells with T_RM_ phenotype suppress tumor growth. Immune checkpoint inhibitors reinvigorate T cells that have lost anti-tumor activity due to immune-inhibitory signaling. **D**) After kidney transplantation, donor-derived T_RM_ cells remain in the graft at least for several months. However, in case of graft rejection, recipient-derived T_RM_ cells replace donor-derived T_RM_ cells and damage the kidney by producing cytokines and cytotoxic molecules

T cells can respond to peptides from pathogens [21]. One of the most common infections in the kidney is bacterial urinary tract infection, affecting young children and adults alike [52–54]. Urinary tract infection is often caused by gut bacteria. *Escherichia coli* is responsible for more than 80% of urinary tract infections in immunocompetent individuals [52–54]. In mouse studies, pyelonephritis induced by *E. coli* results in a significant increase in the number of renal T_RM_ cells [18] (Fig. 1A). Other pathogens such as *Staphylococcus aureus* and *Candida albicans* are also clinically relevant and cause kidney infection in humans. Murine studies demonstrate that renal T_RM_ cells are induced after infection with these pathogens [18]. T_RM_ cells induced by either bacterial or fungal infection show hallmarks of Th17 cells, including high RORγt expression and IL-17A production upon activation. Although T_RM_ cells can provide protection in a variety of infections [51], it is currently unclear to which extent pathogen-specific T_RM_ cells contribute to the host defense against recurrent renal infections.

Kidneys are also a target of viruses such as cytomegalovirus (CMV) or polyomaviruses. These viruses establish a latent, seemingly asymptomatic infection in immunocompetent individuals. However, in immune-compromised patients, including transplant recipients, the immune surveillance is weakened, causing reactivation of viruses and severe systemic illness. CMV infects various types of cells, occasionally also tubular epithelial cells, endothelial cells, and glomerular epithelial cells [55]. In murine CMV infection models, virus-specific T_RM_ cells are induced in many organs but are found at high frequency in the kidney [56, 57]. Polyomavirus is another clinically relevant virus, establishing latent infection in urothelial and renal tubular epithelial cells in a vast majority of the immunocompetent population [58]. In immunosuppressed renal transplant recipients, however, these viruses are reactivated in up to 60% of patients and cause serious conditions such as polyomavirus BK (PyVBK)-associated interstitial nephritis in 10% of the reactivation cases [58, 59]. Polyomavirus-specific T cells were detected in the peripheral blood of healthy individuals [60], in kidney transplant recipients, and even in kidney allografts [59, 60]. PyVBK-specific CD8^+^ T cells in allograft kidney tissue expressed CD69 and CD103 in line with a T_RM_ phenotype. In allografts affected by PyVBK-associated interstitial nephritis, virus-specific CD8^+^ T_RM_ cells were significantly enriched compared with blood [59]. These findings suggest that T_RM_ cells respond and expand after reactivation of the virus, although their activity may not be strong enough to resolve the infection in these patients. It remains unclear how PyVBK-specific T_RM_ cells act after intermittent reactivation in immunocompetent individuals and how they contribute to the control of polyomavirus infection.

## TRM cells in autoimmune kidney diseases

The efficient reactive capacity of T_RM_ cells can be important in the development and/or recurrence of autoimmune diseases. Of note, the tissue manifestation of immune-mediated inflammatory diseases, such as psoriasis, often reoccurs in the same location upon relapse. In previous studies, the presence of T_RM_ cells in the psoriatic skin was described, even during remission, indicating that psoriasis remission and relapse are orchestrated by T_RM_ cells [61–63].

The kidney is a common target organ in relapsing and recurrent autoimmune diseases. In kidneys of patients with ANCA vasculitis or lupus nephritis, a significant number of T cells are observed. Here, it is important to note that the number of T cells in the kidney correlates with disease activity, as indicated by increased serum creatinine, proteinuria, and histological score in patients with lupus nephritis, anti-neutrophil cytoplasmic antibody (ANCA)-associated vasculitis, anti-glomerular basement membrane (GBM) glomerulonephritis [64–66], and as demonstrated in murine lupus models [67]. T cells are found mainly in periglomerular and interstitial regions and less abundantly in intraglomerular regions of the inflamed kidney [64–66]. While CD4^+^ T cells dominate in number compared with CD8^+^ T cells in murine glomerulonephritis models, nearly equal numbers of CD4^+^ and CD8^+^ T cells in humans [64–66, 68] are reported in many studies.

Most of the renal T cells in inflammatory conditions show a tissue-resident phenotype. CD69 is widely expressed in both CD4^+^ and CD8^+^ T cells in the kidney [18, 67, 69]. There are also reports of CD103 expression by CD8^+^ cells in the kidneys of lupus patients and lupus-prone mice [67]. Moreover, molecules such as CCR7, which are important for the homing to lymphatics organs, were not detected in renal T cells from glomerulonephritis patients [18]. In the recent years, single-cell RNA sequencing (scRNAseq) has advanced our understanding of the cellular landscape of immune cells in the kidney. Especially, scRNAseq technology, combined with epitope measurement using barcoded antibodies [cellular indexing of transcriptomes and epitopes by sequencing (CITE-seq)], allowed single-cell transcriptome analysis with surface molecule expression information to be performed. This is of great importance to the tissue-resident immunity analysis because there can be a significant discrepancy between mRNA expression and protein expression of surface markers such as CD69 [18]. Recently, using combined scRNAseq and CITE-seq methods, we have demonstrated the presence of CD4^+^ T_RM_ cells in patients with ANCA-associated glomerulonephritis (GN). In these ANCA patients, we found an increased number of T_RM_ cells compared with healthy kidneys. In addition, transcriptome analysis showed that T_RM_ cells from ANCA patients have a higher proliferative capacity than those isolated from healthy kidneys. The scRNAseq analysis revealed heterogeneity in the tissue-resident CD4^+^ T_RM_-cell population, showing three clusters with different transcriptomic profiles. Another study of scRNAseq analysis of CD45^+^ leukocytes from lupus patients’ kidneys also showed the heterogeneity of T-cell populations, with a distinct CD8^+^ T_RM_-cell cluster expressing the T_RM_-associated proteins Hobit and CD103 [70]. Likewise, it is noteworthy that T cells can be found in the urine of patients. Protein expression profiling of cells from lupus patients’ urine revealed the presence of T cells positive for T_RM_-associated molecules such as CD69 and CD38 [71], which suggests that T cells in the urine originate from T_RM_ cells in the kidney.

**Fig. 2:**
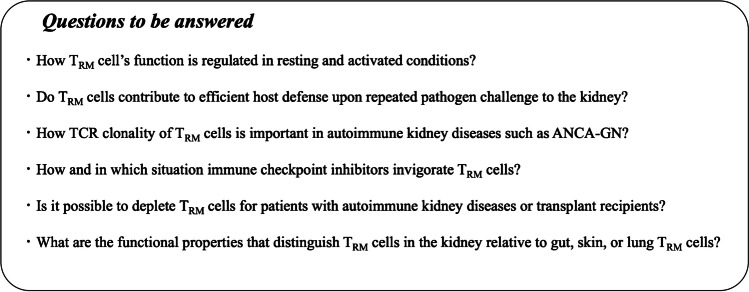
Unanswered questions about TRM cells in kidney diseases

In ANCA glomerulonephritis, the number of T_RM_ cells in the kidney biopsy shows a positive correlation with impaired kidney function as indicated by serum creatinine [18]. Currently, it is unclear how autoantigen-specific T_RM_ cells in the kidney are associated with autoimmune kidney disease development and/or relapse in humans. One possibility is that autoantigen-specific T_RM_ cells are more clonally expanded, especially in relapsing patients, but this assumption needs to be verified by TCR specificity and clonality analysis (Fig. 2). Recently, we have shown that T_RM_ cells induced by *S. aureus* infection aggravates murine crescentic glomerulonephritis [18]. The activation of these T_RM_ cells is probably independent of TCR because they are thought to be mostly *bystander T*_*RM*_* cells*, which express polyclonal TCR non-specific to the kidney antigen. We demonstrated that the innate inflammatory cytokines IL-1β, IL-6, and IL-23 activate T_RM_ cells, leading to cytokine production [18] (Fig. 1B). Most important, T_RM_-cell depletion prevented disease aggravation. These findings suggest that pathogenic T_RM_ cells may be a potential target for the treatment of autoimmune kidney diseases. Targeting their specific survival requirements, such as metabolic reliance on free fatty acids or on IL-15, is currently explored to achieve the depletion of T_RM_ cells [42, 72].

## Kidney tumors and TRM cells

The kidney is a common cancer site, and different types of cancers can develop. The most common kidney cancer, renal cell carcinoma (RCC), is an immunogenic tumor. Here, increased numbers of T cells are observed in the tumor tissue compared with those found in adjacent healthy tissue [11, 73]. In RCC tumor tissue, CD8^+^ T cells are more common [74]. Similar to renal T cells in other diseases, the majority of T cells detected in the tumor show a tissue-resident phenotype with CD69 expression in both CD4^+^ and CD8^+^ T cells [75], and CD103 expression mainly in CD8^+^ T cells [11, 76]. In a mouse renal adenocarcinoma model, most of the CD69^+^ T cells are protected from intravascular staining, supporting the assumption that these T cells are tissue resident.

While a high number of tumor-infiltrating T cells are usually associated with a better prognosis in many tumor types [77], the abundance of intratumoral T cells might be associated with a high tumor grade and shorter patient survival in clear cell RCC (ccRCC) [78]. However, the number of CD103^+^ T cells was reported to be an independent favorable prognostic factor in RCC patients [79]. Furthermore, in a mouse RCC model, CD103^+^-cell depletion resulted in accelerated tumor growth [79]. CD103^+^ T cells are able to respond to TCR reactivation by upregulating cytotoxic mediators and proliferation, suggesting that CD103^+^ CD8^+^ T cells can rapidly reactivate their effector functions [79]. Therefore, CD103^+^ T_RM_-like cells in tumors substantially contribute to immune surveillance. Their potential of immediate anti-tumor responses makes tumor-infiltrating T_RM_ cells an attractive target for therapeutic interventions (Fig. 1C).

The regulation of intratumoral T cells is of great importance to their anti-tumor response. T-cell functionality can be regulated by immune checkpoint proteins such as PD1 and CTLA-4. T cells regulated by inhibitory molecules are unable to proliferate, or to produce cytokine or cytotoxic molecules in response to antigen recognition. The inhibitory protein PD1 was detected on tumor-infiltrating CD103^+^ T cells in RCC, suggesting that their anti-tumor function is actively suppressed and that these tumor-infiltrating T cells can be targeted by checkpoint inhibitors [80]. It is reported that a high proliferation rate of tumor-infiltrating CD8^+^ T cells is associated with prolonged survival of RCC patients. Furthermore, a murine study demonstrated the inefficacy of checkpoint inhibitors after depletion of CD103^+^ cells in a RCC model [79].

Immune checkpoint inhibitors have revolutionized cancer treatment. Clinical trials have provided evidence of a superior survival rate of metastatic RCC patients treated with immune checkpoint inhibitors. Therefore, immune checkpoint inhibition has become a standard of care for advanced RCC [73, 81]. Immune checkpoint inhibition therapy is thought to reinvigorate tumor-infiltrating T cells that have lost their anti-tumor activity due to immune-inhibitory signaling. However, it remains to be investigated how T_RM_ cells in kidney tumors suppress tumor growth. In melanoma models, it is reported that the anti-tumor effect is due to the direct killing of tumor cells, in addition to the activation and recruitment of other immune cells by cytokine production [82, 83].

While many patients benefit from immune checkpoint inhibition therapy, some do not respond to it. Therefore, reliable markers are needed to predict the therapeutic response and to ensure that individuals with a good chance of response receive treatment. Expression of PDL1, a ligand for PD1, has been investigated as a marker of response. However, a clinical response to PD1 inhibition was observed even in patients with low or no PDL1 expression (73, 84). Genetic analysis as a useful predictive tool, such as tumor mutation profiling, was also reported. Accumulating mutations in tumors lead to the formation of increased neoantigens, stimulating immune responses. An analysis of five clinical trials revealed that patients with different tumors such as RCC, with microsatellite instability or deficient mismatch repair, showed a higher response to pembrolizumab, a humanized antibody targeting PD1 [85]. By contrast, another study showed that tumor mutations and neoantigen expression were not associated with progression-free survival of patients treated with anti-PD-L1 [86]. Therefore, further studies addressing the question of how tumor-infiltrating T cells are regulated need to be performed.

In addition to immune checkpoint inhibitors, another innovative approach, namely therapeutic cancer vaccination, has been developed and is being tested. It is reported that the induction of T_RM_ cells enhances the efficacy of cancer vaccines [87]. These vaccines are evolving by the development of next-generation sequencing, which makes it possible to reveal mutations present in tumor cells. By using tumor neoantigens produced by the mutations, cancer vaccines induce T-cell responses specific to cancer cells. First clinical trials showed a robust tumor-specific immunogenicity in patients with melanoma and other cancers. This therapeutic strategy is currently being tested in combination with immune checkpoint inhibitors in a clinical trial for RCC [88].

## Kidney transplantation and TRM cells

Kidney transplantation is an important treatment for patients with end-stage renal diseases. Although the transplantation outcome was significantly improved by the development of immunosuppressants, the rejection of transplanted kidneys remains a serious problem. The graft rejection is caused by T cells. Kidney allografts contain donor-derived T cells, most of which express tissue residency-associated markers such as CD69 and CD103. In the case of HLA-mismatched transplantation, donor- and recipient-derived T cells can be distinguished by HLA staining, making it possible to track T-cell chimerism in the transplanted organs. In lung transplantation, donor-derived T_RM_ cells persist in the organ and express signature markers including CD69, CD103, and CD49a, but the number of donor-derived T cells in the blood becomes negligible at two months following transplantation. Of particular note, recipients with an increased number of donor-derived lung T_RM_ cells have fewer adverse events such as primary graft dysfunction, or acute cellular rejection, compared with recipients with lower donor T_RM_-cell persistence. In kidney transplantation, it is still unclear how long donor-derived cells persist in the graft. A study analyzed transplanted kidneys from patients with graft failure and reported that donor-derived T_RM_ cells were not detected in organs that failed > 5 months after transplantation. This suggests that recipient-derived T cells are recruited to the transplanted kidney, become T_RM_ cells, and replace donor-derived T_RM_ cells when graft failure occurs [12] (Fig. 1D).

Recipient-derived T cells play an important role in the rejection of a transplanted kidney. Both CD4^+^ and CD8^+^-recipient T cells are found in the graft. Based on the expression of cytotoxic molecules such as granzymes A and B, the tissue injury mechanism in T cell-mediated rejection was once thought to be direct cell damage to donor cells. However, tubulitis after allogenic transplantation was observed even in mice deficient in perforin or in the granzymes A and B [89]. Furthermore, a recent study showed that T_RM_ cells in kidney allografts did not express higher amounts of granzyme B, or perforin, compared with circulating cells [12, 17]. Therefore, T cell-mediated rejection is considered to take place through cytotoxic and non-cytotoxic functions [90].

Allo-responsive T cells in recipients can be identified by the detection of cytokine production after stimulation with donor-derived cells. An increased frequency of allo-specific T cells in the peripheral blood of recipients prior to or during the first six months after renal transplantation was associated with an increased risk of acute rejection and inferior graft function [91, 92]. In a mouse model of kidney transplantation, graft rejection was observed only when kidney antigen-specific CD8^+^ T cells were transferred to recipient mice. After transplantation, both kidney antigen-specific and polyclonal T cells were recruited to the graft and differentiated into T_RM_ cells. T_RM_ cells proliferated locally and produced IFN-γ upon re-stimulation with allogenic donor splenocytes (93). Therefore, in kidney transplant rejection, allo-specific T cells in recipients are recruited to the graft, develop into T_RM_ cells, and damage the kidney via production of cytokine and cytotoxic molecules. During this rejection process, host-derived T_RM_ cells are replaced by recipient T_RM_ cells.

## Conclusions

The discovery of T_RM_ cells has significantly advanced our understanding of immunity. Because of their tissue-residing and memory phenotypes, they can respond quickly and efficiently to invading pathogens in the affected organs. The efficient induction of T_RM_ cells in barrier organs would be an attractive strategy for vaccine-mediated immunization against pathogens. Yet, the presence of T_RM_ cells might cause adverse events such as autoimmune disease development and relapse, which makes the induction of T_RM_ cells a double-edged sword. Therefore, it will be important to address the question of how T_RM_-cell activity is regulated and can be targeted for treatment. (Unanswered questions are listed in Fig. 2.)
